# Retaining Providers with Women’s Health Expertise: Decreased Provider Loss Among VHA Women’s Health Faculty Development Program Attendees

**DOI:** 10.1007/s11606-022-07575-5

**Published:** 2022-08-30

**Authors:** Amy H. Farkas, Sarah Merriam, Susan Frayne, Lisa Hardman, Rachel Schwartz, Christine Kolehmainen

**Affiliations:** 1 Division of General Internal Medicine, Medical College of Wisconsin, Milwaukee, WI USA; 2 Department of Medicine, Milwaukee VA Medical Center, Milwaukee, WI USA; 3 Office of Women’s Health, Veterans Health Administration, Washington, DC USA; 4 Division of General Internal Medicine, University of Pittsburgh School of Medicine, Pittsburgh, PA USA; 5 Department of Medicine, VA Pittsburgh Healthcare System, Pittsburgh, PA USA; 6 VA HSR&D Center for Innovation to Implementation (Ci2i), VA Palo Alto Health Care System, Menlo Park, CA USA; 7 Division of Primary Care and Population Health, Stanford University School of Medicine, Stanford, CA USA; 8 WellMD Center, Stanford University School of Medicine, Stanford, CA USA; 9 Division of Internal Medicine, University of Wisconsin School of Medicine and Public Health, Madison, WI USA

**Keywords:** women’s health, faculty development programs, retention

## Abstract

**Background:**

The Veterans Health Administration (VHA) provides care for over 500,000 women. In 2010 VHA instituted a policy requiring each facility to identify a designated women’s health provider (WH-PCP) who could offer comprehensive gender-specific primary care. Access to WH-PCPs remains a challenge at some sites with high turnover among WH-PCPs. Faculty development programs have been demonstrated to foster professional development, networks, and mentorship; these can enhance job satisfaction and provide one potential solution to address WH-PCP turnover. One such program, the VHA’s Women’s Health Mini-Residency (WH-MR), was developed in 2011 to train WH-PCPs through case-based hands-on training.

**Objective:**

The objective of this program evaluation was to determine the association of WH-MR participation with WH-PCP retention.

**Design:**

Using the Women’s Health Assessment of Workforce Capacity-Primary Care survey, we assessed the relationship between WH-MR participation and retention of WH-PCP status between fiscal year 2018 and 2019.

**Participants:**

All WH-PCPs (*N* = 2664) at the end of fiscal year 2018 were included.

**Main Measures:**

We assessed retention of WH-PCP status the following year by WH-MR participation. For our adjusted analysis, we controlled for provider gender, provider degree (MD, DO, NP, PA), women’s health leadership position, number of clinical sessions per week, and clinical setting (general primary care clinic, designated women’s health clinic, or a combination).

**Key Results:**

WH-MR participants were more likely to remain WH-PCPs in FY2019 in both unadjusted analyses (OR 1.91, 95%CI 1.54–2.36) and adjusted analyses (OR 1.96, 95%CI 1.58–2.44).

**Conclusions:**

WH-PCPs who participate in WH-MRs are more likely to remain WH-PCPs in the VHA system. Given the negative impact of provider turnover on patient care and the significant financial cost of onboarding a new WH-PCP, the VHA should continue to encourage all WH-PCPs to participate in the WH-MR.

## BACKGROUND

In 2010, Veterans Health Administration (VHA) remodeled care for women Veterans and designated women’s health primary care providers (WH-PCPs) to preferentially offer comprehensive gender-specific care.^1,2^ Women Veterans who receive care from WH-PCPs are more likely to receive gender-specific cancer screening,^3^ use contraception,^4,5^ report screening for military sexual trauma and intimate partner violence,^6^ and endorse higher levels of satisfaction with care.^7^

Although VHA has expanded women-centric healthcare coverage, WH-PCP attrition (14% per year) continues to present a barrier to widespread provision of gender-specific care to women Veterans at some sites.^8^ Additionally, the attrition rate among WH-PCPs is higher than for physicians overall within VHA (average annual turnover rate of approximately 9.5%)^9^. While the reasons for such high attrition are not fully understood, WH-PCPs do require distinct skills to care for a medically complex patient population in a system that has historically been focused on men.^10,11^ Additionally, WH-PCPs are expected to maintain medical expertise through additional continuing educational requirements beyond those required of physicians without the WH-PCP designation^2^.

Unfortunately, provider turnover negatively impacts the patient experience in the general population,^6^ and the same holds true for women Veterans. For example, in 2020, women Veterans identified a lack of regular access to a WH-PCP as a barrier to reproductive healthcare within the VHA.^12^ Such barriers to care almost certainly impact the overall health and well-being of women Veterans.

Faculty development programs (FDPs) provide one potential solution to address challenges in WH-PCP recruitment and retention. Physicians value FDPs,^13^ and continuing medical education programs improve physician practices and patient outcomes.^14^ FDPs also foster the development of professional networks, social support, and mentorship which can enhance job satisfaction and commitment to the institutional mission.^15,16^ VHA Office of Women’s Health currently offers a few FDPs aimed at improving women’s health knowledge and clinical skills of primary care providers. Of these programs, the Women’s Health Mini-Residency (WH-MR) national training program is the largest and most intensive. The WH-MR is one mechanism through which VHA providers can become designated WH-PCPs.

The WH-MR began in 2008 and has trained 4820 providers since its inception (internal program data). The program offers national trainings each summer: providers from around the country join for 3 days of case-based educational programming. In addition to large group didactic seminars, the WH-MR provides opportunities for facilitated small group case discussion and knowledge application and also simulated gynecological skills practice using both breast and pelvic models and live simulated patients. Each year approximately 500 primary care providers including physicians, nurse practitioners, and physician assistants attend the program, along with 40 teaching faculty. The WH-MR has been highly rated by participants in terms of satisfaction with the program and improvement in comfort with the clinical care of women Veterans (internal program evaluation data). However, the impact of WH-MR on WH-PCP retention remains unknown.

Therefore, the objective of this program evaluation was to determine the impact of WH-MR participation on WH-PCPs retention.

## METHODOLOGY

### Design

We assessed the relationship between WH-MR participation and retention of WH-PCP status between FY2018 and 2019. Our analysis used data from the Women’s Health Assessment of Workforce Capacity-Primary Care survey (WAWC), an annual survey of VHA WH-PCPs conducted at the end of each federal fiscal year (FY) by Women Veteran Program Managers, a central liaison for women’s health at each VHA medical facility.^17^ The survey has a 100% response rate as the Women Veteran Program Managers are required to complete it for each WH-PCP.

### Participants

We included all VHA WH-PCPs nationally who were medical doctors/doctors of osteopathy (MDs/DOs), nurse practitioners (NPs), or physician assistants (PAs). We excluded fellows and those providers who carried the WH-PCP designation but were not active primary care providers (absence of patient panels and/or clinic sessions). Individuals who attended the WH-MR for the first time in FY2019 were excluded from the cohort as the impact of participation in the WH-MR could not be assessed over such a short timeframe. Individuals who retired in FY2019 were also excluded from our analysis of retention as this reason for departure was determined to be different from other factors contributing to turnover.

### Outcome Variable: Retention of WH-PCP Status

**Figure 1:**
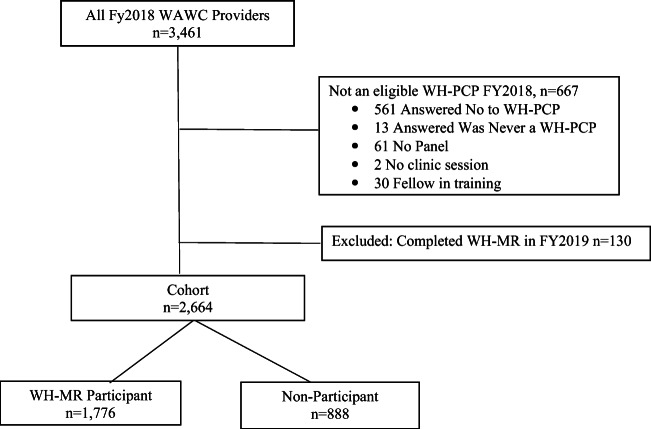
Cohort development process.

We used data from the FY2018 WAWC survey to identify WH-PCPs at the end of September 30, 2018, and data from the FY2019 survey to assess retention. To confirm that each WH-PCP maintained an active primary care panel of patients, WAWC data were linked with the Patient Care Management Module, in national VHA administrative data (Fig. 1). This work received a Determination of Non-Research from VHA Central Office and was funded as program evaluation by VHA Women’s Health.

### Data Analysis

In our logistic regression analysis, we examined retention of WH-PCP status (retention coded as 1 and all attrition coded as 0) as a function of WH-MR participation. For our multivariable adjusted analysis, we controlled for provider gender, provider degree (MD, NP, PA), women’s health leadership position, number of clinical sessions per week, and clinical setting (general primary care clinic, designated women’s health clinic, or a combination). Stata SE was used for all statistical calculations.

## KEY RESULTS

Our cohort included 2664 WH-PCPs in FY2018. The majority of WH-PCPs, 1984 (74.5%), were women and 1856 (69.7%) were physicians, 669 (25.1%) were nurse practitioners, and 139 (5.2%) were physician assistants. Two-thirds (1776) had previously attended the WH-MR. WH-MR participants were more likely to be female, non-physicians, and work in a designated women’s health clinic (Table 1).

**Table 1:** Characteristics of Veteran Health Administration (VHA) Designated Women’s Health Primary Care Providers

Variable	WH-MR participant *n* = 1776	Non-participant *n* = 888	*p* value
Gender, *n* (%)			< 0.001
Female	1393 (78.4)	591 (66.6)	
Male	383 (21.6)	297 (33.5)	
Provider degree, *n* (%)			< 0.001
Physician	1195 (67.3)	661 (74.4)	
Nurse practitioner	489 (27.5)	180 (20.3)	
Physician assistant	92 (5.2)	47 (5.3)	
Women’s health leadership position, *n* (%)	315 (17.7)	241 (27.1)	< 0.001
Clinical half-day sessions per week, mean (SD)	8.1 (2.7)	7.8 (2.7)	0.02
Clinic setting, *n* (%)			< 0.001
General primary care clinic	1476 (83.1)	795 (89.5)	
Combination	96 (5.4)	38 (4.3)	
Designated women’s clinic	204 (11.5)	55 (6.2)	

Of the original cohort, 2155 (80.9%) retained the WH-PCP designation status in FY2019, yielding an attrition rate of 19.1% (Table 2). After excluding individuals who had retired between FY2018 and FY2019, 86.7% of WH-MR participants remained WH-PCPs in FY2019 compared to 77.4% of non-participants (*p* < 0.001) (Table 2). After controlling for provider gender, provider degree (MD/NP/PA), women’s health leadership positions, clinical sessions per week, and clinical setting, WH-MR participants were more likely to remain WH-PCPs in FY2019 in both unadjusted analyses (OR 1.91, 95%CI 1.54–2.36) and adjusted analyses (aOR 1.96, 95%CI 1.58–2.44) (Table 3). Additionally, nurse practitioners were more likely to remain WH-PCPs (aOR 1.52 95%CI 1.18–1.96). Those who practiced in a designated women’s clinic only were less likely to remain WH-PCPs (aOR 0.64 95%CI 0.42–0.99).

**Table 2:** Veteran Health Administration (VHA) Designated Women’s Health Provider (WH-PCP) Attrition Data by Women’s Health Mini-Residency (WH-MR) Participation

Variable	WH-MR participant *n* = 1776	Non-participant *n* = 888	*p* value
Total attrition, *n* (%)	281 (15.8)	228 (25.7)	< 0.001
Reason for attrition, *n* (%)			0.04
Left-VHA	67 (23.8)	63 (27.6)	
Still in VHA not WH-PCP	116 (41.3)	85 (37.3)	
Extended leave	13 (4.6)	3 (1.3)	
Retired	52 (18.5)	35 (15.4)	
Unknown	33 (11.7)	42 (18.4)

**Table 3:** Unadjusted and Adjusted Analysis for Designated Women’s Health Provider (WH-PCP) Retention

	Unadjusted OR (95%CI)^a^ *N* = 2155	Adjusted OR (95%CI)^b^ *N* = 2155
MR participation	1.91 (1.54–2.36)*	1.96 (1.58–2.44)*
Gender	1.05 (0.83–1.34)	1.93 (0.79–1.33)
Provider type		
Physician	Reference	Reference
Nurse practitioner	1.27 (1.08–1.73)*	1.52 (1.18–1.96)*
Physician assistant	1.32 (0.84–2.07)	1.34 (0.84–2.12)
WH leadership position	1.19 (0.91–1.55)	1.23 (0.96–1.68)
Number of clinical half-day sessions per week	0.98 (0.94–1.01)	0.96 (0.93–1.00)
Clinical setting		
General primary care clinic	Reference	Reference
Combination	0.89 (0.55–1.45)	1.01 (0.61–1.67)
Designated women’s clinic	0.58 (0.38–0.87)*	0.64 (0.42–0.99)*

^a^Unadjusted odds ratios displayed in this column reflect unadjusted regression examining retention as a function of the variable listed

^b^Adjusted odds ratios reflect a single multivariable logistic regression controlling for all variables listed

*Statistically significant finding

## DISCUSSION

Women’s Health Primary Care Providers who participate in VHA’s Women’s Health Mini-Residency are more likely to remain WH-PCPs in the VHA system. The protective effect of the WH-MR is consistent with previous literature demonstrating the value of FDPs.^13–16^ This finding is also consistent with data showing VHA providers who perceive themselves and the institution as providing high-quality care report higher job satisfaction and less intention to leave.^18^

Further, taking into consideration both the negative patient outcomes and financial cost of turnover,^19^ FDPs both within and outside of the VHA should consider assessing clinician retention as part of program evaluation data. If found, a positive impact on retention could be utilized as justification of the costs associated with FDPs. The value of dedicated women’s health provider development is increasingly recognized both within and outside VHA. In January 2021, Congress enacted the Johnny Isakson and David P. Roe M.D. Veterans Health Care and Benefits Improvement Act which aims, among other requirements, to increase opportunities for providers to participate in women’s health educational programs including the WH-MR.

Beyond the impact of the WH-MR on retention, our data suggest that NPs are more likely to remain WH-PCPs. This has implications for the WH-MR program in terms of both participant recruitment and course content, particularly given that VHA is the largest employer of NPs and grants NPs full practice authority.^20^ Additionally, our finding that WH-PCPs who practiced solely in designated women’s health clinics had higher attrition rates is inconsistent with previous data.^8^ While this may be in part related to the different time frame of this study, further analysis will be needed to reconcile these results. The VHA should consider additional investigation to better define which specific aspects of the clinical environment most impact provider retention.

This study does have limitations. Given the retrospective study design and cross-sectional nature of these data, it is not possible to determine causation. It is possible that additional, unaccounted for factors may have impacted the differential rates of retention observed. Additionally, we cannot exclude a selection bias, wherein providers with high dedication to women’s health were more likely to attend the WH-MR and thus more likely to remain a WH-PCPs after participation. Qualitative analysis could help to delineate both the perceived benefits of the WH-MR and the underlying reasons driving attendance and participation. Further, as WAWC survey data is reported by Women Veteran Program Managers, we cannot exclude the possibility of misclassification of WH-PCP status and/or WH-MR attendance designation. Finally, this study does not explore the impact of virtual faculty development on provider retention. It is unclear how the transition from face-to-face to virtual programming in the context of the COVID-19 pandemic may have impacted the protective effect of faculty development on retention, especially given that many purported benefits relate to developing shared mission, networking, mentorship, and the development of social supports.^15,16^ While virtual programming tends to be less expensive than traditional face-to-face programming and eliminates, in part, time away from clinical duties, the saved expenses may be negated if the virtual format does not offer equivalent benefits in terms of retention.

As the population of women Veterans continues to grow, the WH-MR offers one potential mechanism through which the VHA can help ensure access to a well-trained WH-PCPs workforce in keeping with an overarching commitment to providing excellent care to all. Future work should explore the perceived benefits of WH-MR participation to help clarify mechanisms for the program’s impact on provider retention. Additionally, other FDPs both within and outside of the VHA should consider assessing impact on provider retention as a marker of effectiveness, potentially to include conduct an analysis of costs associated with recruitment and onboarding compared with those of faculty development.

## CONCLUSION

Given the high costs of recruitment and on-boarding of new providers, which ranges from $250,000 to $1 million depending on the provider,^21,22^ the VHA should encourage WH-PCPs to participate in the WH-MR program as a potential mitigation strategy for high rates of WH-PCP attrition. Additionally, further evaluation of the impact of the WH-MR program, to include a more longitudinal or qualitative analysis, is needed to elucidate the perceived benefits of this FDP on retention.
